# C-section technique vs minilaparotomy after minimally invasive uterine surgery: a retrospective cohort study

**DOI:** 10.1007/s00404-023-07239-7

**Published:** 2023-10-05

**Authors:** Luigi Della Corte, Maria Chiara Guarino, Salvatore Giovanni Vitale, Stefano Angioni, Antonio Mercorio, Giuseppe Bifulco, Pierluigi Giampaolino

**Affiliations:** 1 Department of Neuroscience, Reproductive Sciences and Dentistry, School of Medicine, University of Naples Federico II, 80131 Naples, Italy; 2 Department of Public Health, University of Naples Federico II, 80131 Naples, Italy; 3 Division of Gynecology and Obstetrics, Department of Surgical Sciences, University of Cagliari, 09124 Cagliari, Italy

**Keywords:** C-section technique, Minilaparotomy, Myomectomy, Laparoscopy, Pain management

## Abstract

**Purpose:**

Uterine leiomyomas are benign uterine tumors. The choice of surgical treatment is guided by patient's age, desire to preserve fertility or avoid "radical" surgical interventions such as hysterectomy. In laparotomy, the issue of extracting the fibroid from the cavity does not arise. However, in laparoscopy and robotic surgery, this becomes a challenge. The aim of the present study was to determine the optimal surgical approach for fibroid extraction following laparoscopic or robotic myomectomy in terms of postoperative pain, extraction time, overall surgical time, scar size, and patient satisfaction.

**Methods:**

A total of 51 patients met the inclusion criteria and were considered in our analysis: 33 patients who had undergone the "ExCITE technique" (Group A), and 18 patients a minilaparotomy procedure (Group B), after either simple myomectomy, multiple myomectomy, supracervical hysterectomy, or total hysterectomy. The diagnosis of myoma was histologically confirmed in all cases.

**Results:**

Regarding the postoperative pain evaluation, at 6 h, patients reported 4 [3–4] vs 6 [5.3–7] on the VAS in Group A and B, as well as at 12 h, 2 [0–2] vs 3.5 [2.3–4] in Group A and B, respectively: both differences were statistically significant (*p* < 0.001). No statistically significant difference at 24 h from surgery was found. All patients in Group A were satisfied with the ExCITE technique, while in Group B only 67% of them. The length of the hospital stay was significantly shorter in Group A as compared to Group B (*p* = 0.007). In terms of the operative time for the extraction of the surgical specimen, overall operative time, and the scar size after the surgery, there was a statistically significant difference for those in Group A.

**Conclusion:**

The ExCITE technique does not require specific training and allows the surgeon to offer a minimally invasive surgical option for patients, with also an aesthetic result. It is a safe and standardized approach that ensures tissue extraction without the need for mechanical morcellation.

## What does this study add to the clinical work

**Table:** 

The ExCITE technique does not require specific training and allows the surgeon to offer a minimally invasive surgical option for patients, with also an aesthetic result.
The ExCITE technique is a safe and standardized approach that ensures tissue extraction without the need for mechanical morcellation.

## Introduction

Uterine leiomyomas, also known as fibroids, are non-cancerous, steroid-dependent tumors that arise from the myometrium [1]. Myomas typically exhibit slow growth, although if there is a rapid increase in size, it can be considered a red flag for possible malignancy, such as sarcoma [1]. While the normal myometrium has a limited response to estrogen, myoma shows increased expression of estrogen-regulated genes and estrogen receptors as well as growth capacity in response to progesterone [2, 3]. Myomas are the most common benign tumors found in women of reproductive age, and they have a significant impact on their quality of life [4]. The main symptoms include dysmenorrhea, pelvic discomfort, menorrhagia, urinary incontinence, anemia, recurrent miscarriages, premature birth, and, in some cases, infertility [4]. The diagnosis is made through ultrasound examination, magnetic resonance imaging and/or hysteroscopy, in particular for those intramural myomas with an important submucosal component [5–7]

Medical treatment includes the use of estroprogestins, progestins, or GnRH analogs [8, 9]. The choice of a surgical treatment is guided by patient’s age, desire to preserve fertility or avoid “radical” surgical interventions such as hysterectomy. Based on the location, the main treatments include hysteroscopic myomectomy, laparotomic or laparoscopic/robotic myomectomy. Currently, the indications for open myomectomy include large uterine fibroids (> 10cm) causing heavy menstrual bleeding and pelvic pain, as well as cases of multiple fibroids [10]. The laparoscopic approach is increasingly being used due to its advantages in reducing postoperative complications and hospital stays. The benefits appear to be further enhanced with the robotic approach, although it is clearly more expensive and time-consuming. Women with more than four fibroids in different locations, large fibroids (> 8–10 cm), fibroids located below the uterine ligaments, or limited uterine mobility due to previous surgeries should be excluded from the laparoscopic approach [11]. In laparotomy, the issue of extracting the fibroid from the cavity does not arise. However, in laparoscopy and robotic surgery, this becomes a challenge.

There are currently three strategies to overcome this issue. Fistly, after laparoscopy or robotic surgery, to perform extraction of the specimen, with the minilaparotomy surgeon makes an abdominal incision of no more than 6–7 cm, usually 3–5 cm above the pubic area or in the lower abdomen [12]. Regarding the mechanical morcellation, the fibroid is sectioned into multiple parts in the abdominal cavity. However, since 2014, the FDA has advised against this technique due to the risk of disseminating fibroids within the cavity, as they may contain neoplastic cells from an occult leiomyosarcoma. One technique to overcome this issue is to perform morcellation inside an endobag containing the fibroid [13]. Finally, the ExCITE technique (Extracorporeal C-incision tissue extraction) provides a viable alternative to mechanical morcellation. This surgical technique involves an enlargement at the incision site ranging from 2.5 to 3.5 cm and the use of a wall protector/retractor (O-Ring Alexis) inserted into the incision [14]. Based on these considerations, the aim of our study was to gather sufficient evidence to determine the optimal surgical approach for fibroid extraction following laparoscopic or robotic myomectomy in terms of postoperative pain, post-myomectomy fibroid extraction time, overall surgical time, scar size, and patient satisfaction.

## Materials and methods

This was a retrospective observational cohort study carried out at the Gynecological Unit of DAI Materno-Infantile Federico II in Naples, Italy, from January 2022 to March 2023. The study enrolled 51 patients aged between 18 and 65 years old who were diagnosed with uterine fibroids (single or multiple fibroids) and presented with symptoms such as menorrhagia and/or pelvic pain. Exclusion criteria were: age < 18 or > 65 years old, oncological disease, high anesthesiological risk (ASA 3–4), fibroid size larger than 10 cm that did not allow removal through laparoscopy, significantly enlarged uterus reaching the transverse umbilical line and ongoing pregnancy. The patients’medical records were retrieved, and based on screening of the clinical and anamnestic data, those who met the inclusion criteria were included in the study. The diagnosis of uterine fibroids was made using a GE Voluson 730 Ultrasound Machine. In case of need, in particular in the presence of ompression signs on the bladder or bowel and/or important vascularization at ultrasound (Color Score ≥ 3), abdominal and pelvic magnetic resonance imaging (MRI) with and without contrast was performed. Patients underwent either simple myomectomy, multiple myomectomy, supracervical hysterectomy, or total hysterectomy, depending on their clinical needs and surgical recommendations.

Based on clinical and anamnestic criteria, the patients were divided into two groups according to the post-myomectomy extraction technique.

**Fig. 1:**
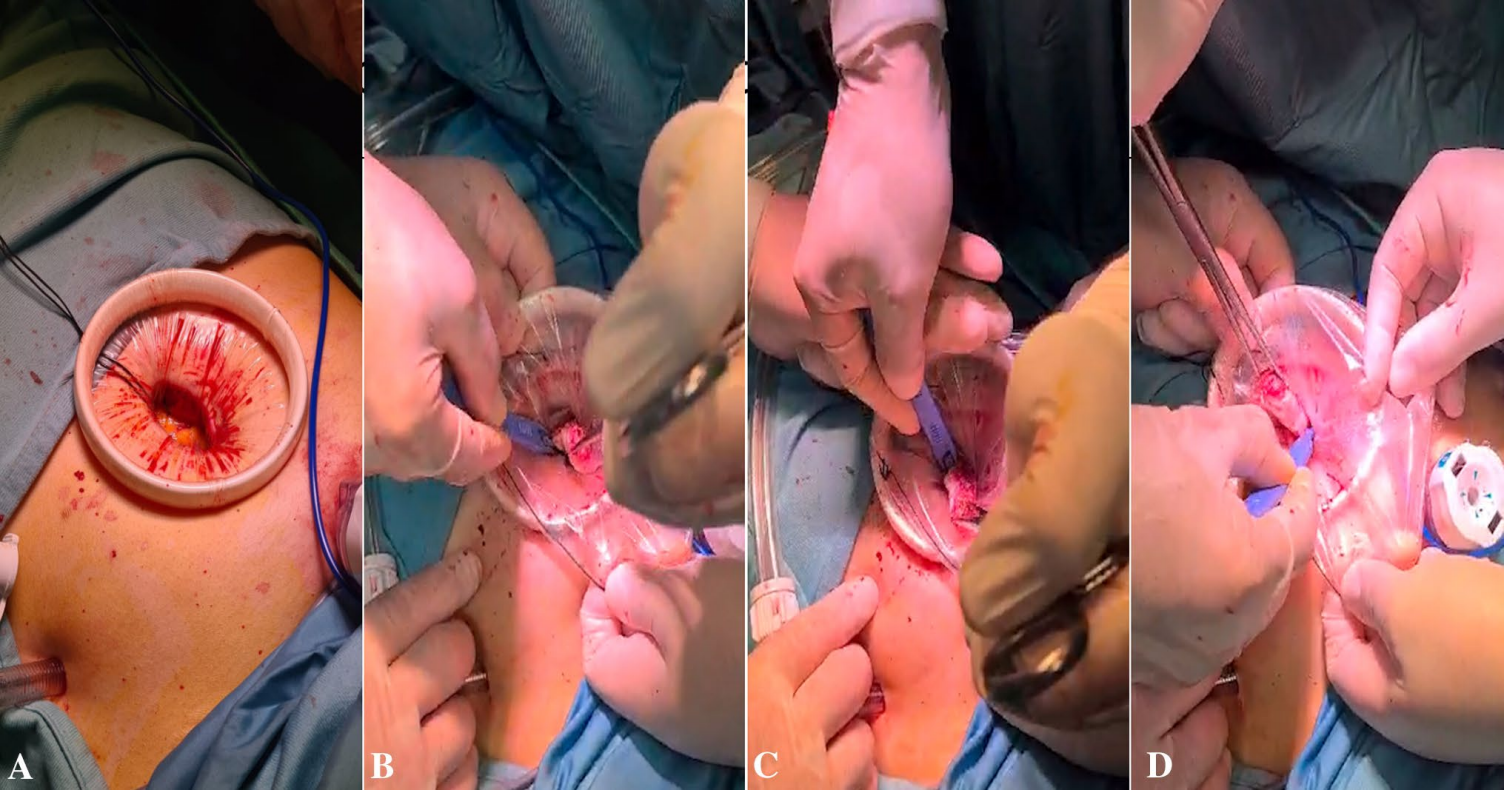
**A–D** The steps of EXCITE technique: from the placement of wall protector/retractor (O-Ring Alexis) to gradual removal of the surgical piece

In 33 patients, the fibroids were extracted using the ExCITE (Extracorporeal Instrument for Tissue Extraction) technique. This type of surgery involves placing the fibroid in an endobag after its enucleation. Subsequently, the incision is enlarged at the umbilicus or at the site of ancillary trocars, widening it to 2.5–3.5 cm. By using a wall protector/retractor (O-Ring Alexis) inserted into the incision, atraumatic 360-degree retraction and protection are provided during the extraction of the specimen, which is kept within the bag near the incision. The fibroid is grasped with forceps and pulled in a way that brings it close to the incision. A scalpel is used to make an initial reverse C-shaped incision in order to facilitate the extraction until the complete removal of the surgical specimen (Fig. 1) [14].

In the remaining 18 patients, the fibroids were extracted through a minilaparotomy.

Pre- and post-operative hemoglobin levels were measured by collecting blood samples. The blood loss during the surgery was measured using a suction device with graduated containers, excluding any washing fluid. This allowed for an estimation of the amount of blood loss during the surgical procedure. According to our internal anesthetic protocol [15, 16], the management of postoperative pain included the administration of Paracetamol 1000 mg (up to three times a day) in case of VAS < 5 and of Ketorolac 30 mg in case of VAS ≥ 5. In case of inadequate analgesia, after 60 min of the Ketorolac after administration, Oxicodone 10 mg i.v. was used. All the samples were histologically analyzed and confirmed the diagnosis of myoma.

### Statistical analysis

Concerning the continuous variables, once the normality assumption was assessed using the Shapiro–Wilk test, the medians, first and third quartiles were reported. To test the hypothesis of equality between the two groups, the Wilcoxon test was performed. For categorical variables, the frequencies and their rates were reported. The ANOVA (Analysis of Variance) test was used as a statistical test. Finally, the distributions of the outcome in relation to the extraction techniques were represented using boxplots. Statistical significance was considered with *p* < 0.05.

## Results

A total of 51 patients met the inclusion criteria and were considered in our analysis: 33 patients underwent the "ExCITE technique" (Group A), while 18 patients underwent a minilaparotomy procedure (Group B). The average age was 40 [34–43] years in Group A and 46 [43–49] years old in Group B. There was a significant difference in BMI between the two groups, greater than in Group A (35.1 [31.0–36.3]) as compared to Group B (31.0 [27.8–32.0]). The variables under examination were adjusted for age and BMI, which were considered confounding factors. All patients were affected by uterine fibroids. The maximum size of the fibroid was 7 [6–8] cm in Group A and 9 [6–9] cm in Group B, showing a significant difference between the two groups (*p* < 0.002). The demographic and clinical characteristics of each group were compared and  reported in Table 1.

**Table 1:** Demographic and clinical characteristics of each group

	Group A (*n* = 33)	Group B (*n* = 18)	*p* value
Age	40 [34–43]	46 [43–49]	< 0.001
BMI	35.1 [31.0–36.3]	31.0 [27.8–32.0]	0.011
Myoma size (cm)	7 [6–8]	9 [6–9]	0.002
Surgical technique			< 0.001
Simple myomectomy	20 (61.0%)	4 (22.0%)	
Multiple myomectomy	12 (36.0%)	3 (17.0%)	
Supracervical or total hysterectomy	1 (3.0%)	11 (61.1%)	
Surgical indication			0.009
Uterine myoma	20 (60.6%)	4 (22.0%)	
Uterine fibroma	13 (39.4%)	14 (78.0%)	

Values are expressed as median, first and third quartile

**Fig. 2:**
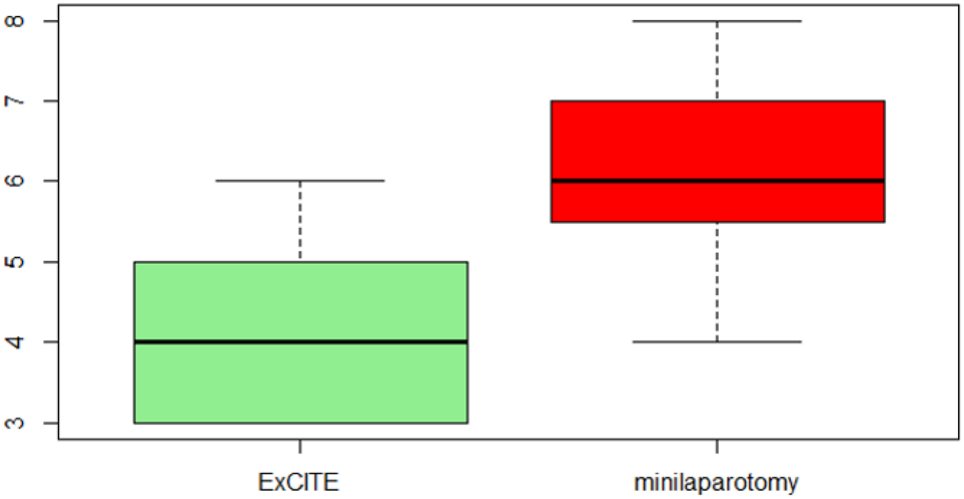
Postoperative pain evaluated through VAS after 6 h from surgery

**Fig. 3:**
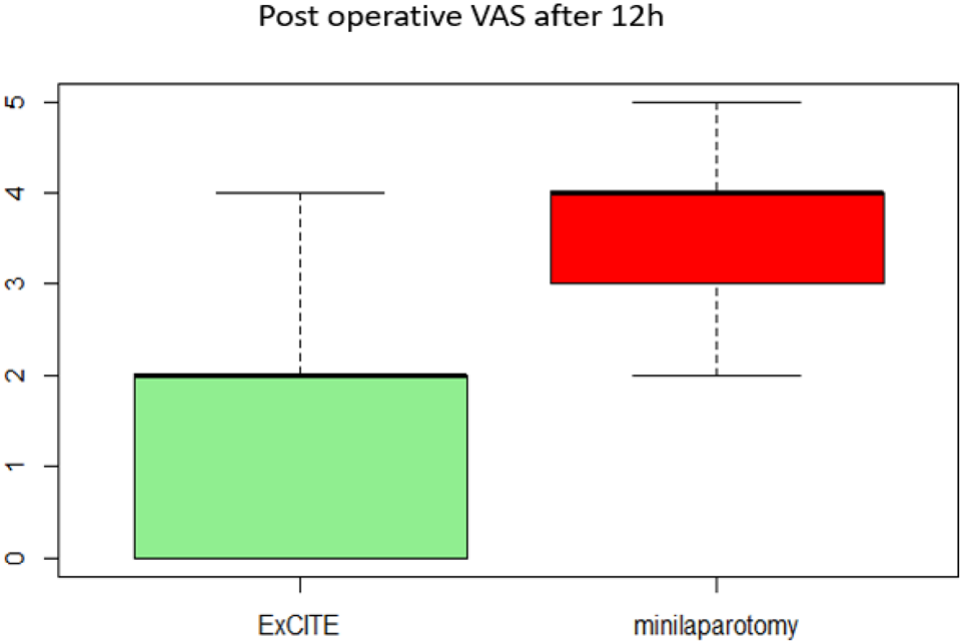
Postoperative pain evaluated through VAS after 12 h from surgery

**Fig. 4:**
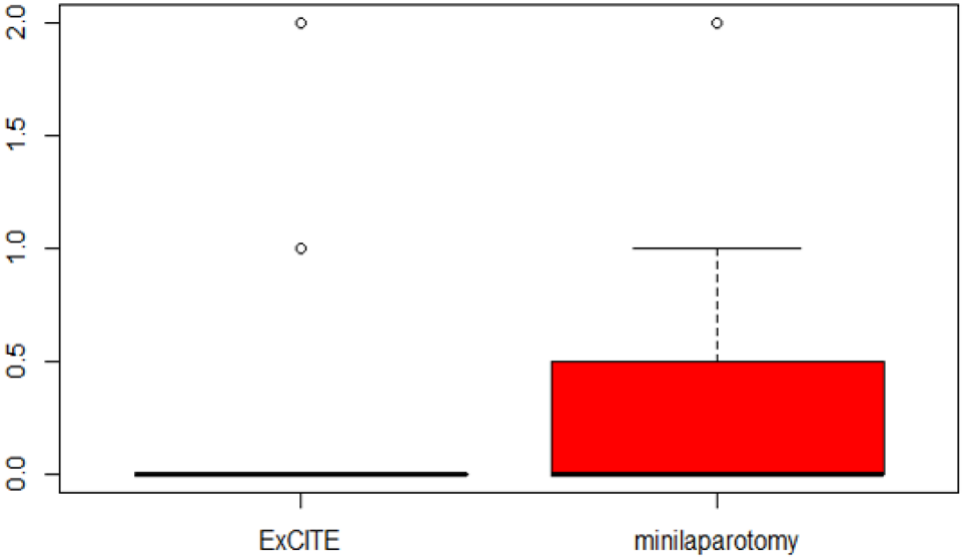
Postoperative pain evaluated through VAS after 24 h from surgery

Regarding the postoperative period, Table 2 illustrates the results of postoperative pain using the VAS (Visual Analog Scale) at 6, 12 and 24 h. At 6 h, patients reported 4 [3, 4] on the VAS in Group A and 6 [5.3–7] in Group B (Fig. 2). At 12 h, it was reported as 2 [0–2] in Group A and 3.5 [2.3–4] in Group B (Fig. 3). Both differences were statistically significant, indicating a higher tolerance for postoperative pain in the ExCITE group. The difference in postoperative pain on the VAS after 24 h of surgery was not found to be statistically significant (Fig. 4).

**Table 2:** Main outcome: assessment of postoperative pain using VAS (Visual Analog Scale).

	Group A (*n* = 33)	Group B (*n* = 18)	*p* value
VAS postoperative pain after 6 h	4 [3, 4]	6 [5.3–7]	< 0.001
VAS postoperative pain after 12 h	2 [0–2]	3.5 [2.3–4]	< 0.001
VAS postoperative pain after 24 h			0.683
0	29 (88.0%)	14 (78.0%)	
1	3 (9.0%)	3 (17.0%)	
2	1 (3.0%)	1 (6.0%)	

Continuous variables are expressed as median, first quartile, and third quartile, while categorical variables are expressed as frequency and percentage

As illustrated in Table 3, while all patients in Group A were satisfied regarding the ExCITE technique, in Group B only 67% of the patients have been. The length of the hospital stay was significantly shorter in Group A as compared to Group B (*p* = 0.007). Table 3 also reports the difference between myoma extraction with the ExCITE technique and minilaparotomy in terms of operative time for the extraction of the specimen, overall operative time, and the scar size after the surgery, all of which turned out to be significantly better with the first procedure. Pre and postoperative hemoglobin levels, as well as estimated blood loss during the surgery, did not show significant differences between the two groups. The ExCITE extraction technique allowed for faster extraction of the surgical specimen, with a median time of 20 [15–20] minutes in Group A and 25 min [20–25] in Group B. It reduced the overall operative time to a median of 75 [65.0–105.0] minutes in Group A vs 93 [81.2–120] minutes in Group B. The scar sizes after surgery were significantly smaller in Group A, with an average of 2 [2] cm, as compared to an average of 7 [7] cm in Group B.

**Table 3:** Secondary outcomes: patient satisfaction, length of hospital stay, operative time for the extraction of the specimen, overall operative time, scar size, pre-postoperative hemoglobin levels and estimated blood loss

	Group A (*n* = 33)	Group B (*n* = 18)	*p* value
Patient satisfaction			< 0.001
Yes	33 (100.0%)	12 (67.0%)	
No	0 (0%)	6 (33.0%)	
Hospital stay (days)	3 [2, 3]	3 [3–3.75]	0.007
Operative time for the extraction (min)	20 [15–20]	25 [20–25]	0.037
Overall operative time (min)	75.0 [65.0–105.0]	93 [81.2–120]	0.032
Scar size (cm)	2 [2–2]	7 [7–7]	< 0.001
Pre operative Hemoglobin (g/dl)	11.1 [10.8–12.1]	12.0 [11.5–12.5]	0.195
Postoperative Hemoglobin (g/dl)	10.8 [10.5–11.9]	11.4 [10.6–11.9]	0.323
Estimated blood loss (ml)	100 [100–150]	115 [100–187.5]	0.278

Continuous variables are expressed as median, first quartile, and third quartile; categorical variables are expressed as frequency and percentage

## Discussion

Uterine fibroids are benign tumors that occur during women’s fertile age and are the leading cause of hysterectomy worldwide. By the age of 50, fibroids have a prevalence of 65%, with a significant increase of up to 90% in Afro-American women. Not all fibroids cause symptoms, but approximately 30% of affected women experience abnormal uterine bleeding, which can lead to anemia, prolonged menstrual periods, pelvic compression symptoms, and reproductive issues. Despite being a benign condition, fibroids represent a chronic disease for many women and also impose significant healthcare costs [17].

We have compared two surgical techniques for extracting fibroids from the abdominal cavity after laparoscopic myomectomy, not only in terms of effectiveness but also satisfying in terms of patient postoperative pain, healing time, scar size, extraction time of the surgical specimen, and overall operative time.

Myomectomy is a conservative surgical treatment that allows for the preservation of the uterus and therefore fertility. It can be performed through different surgical approaches, including laparotomy, laparoscopy/robotic-assisted surgery, or hysteroscopy. The choice of surgical approach primarily depends on the location of the fibroids, their number and their size [18]. When conditions allow, it is clearly preferable to opt for a minimally invasive technique. However, in the case of large-sized fibroids, the challenge with laparoscopy or robotic-assisted surgery arises during the step following specimen excision, which is the removal of the fibroid itself from the abdominal cavity when transvaginal removal is not possible due to its size. The solutions adopted so far have been minilaparotomy and mechanical morcellation. However, since the FDA discouraged the use of mechanical morcellation as a technique for specimen extraction in 2014, due to the risk of developing disseminated peritoneal leiomyomatosis [19, 20], as well as the potential dissemination of malignant cells if it is a uterine sarcoma, there has been a need to find a valid alternative [13].

This is the first study that has compared the ExCITE technique and minilaparotomy as approaches for the extraction of myomas after laparoscopic or robot-assisted myomectomy or hysterectomy that are too large to be directly removed from the abdominal cavity through a laparoscopic incision or transvaginally.

The first study on the ExCITE technique was conducted by Cesta et al. [21] that reported a case of a woman who underwent laparoscopic total hysterectomy (with a uterus weighing 7400 g), bilateral salpingectomy and right ovariectomy. Following the ExCITE technique, the umbilical incision was enlarged up to 4 cm, and the surgical specimens were extracted using morcellation with a surgical scalpel. The patient was then discharged the day after the surgery without any complications.

In a similar study by DAlbuquerque et al. [22], a total hysterectomy was performed on a uterus eight times larger than normal due to diffuse leiomyomatosis. The procedure was safely performed using robot-assisted laparoscopy, and the uterus was removed through a vaginal approach, using morcellation with the ExCITE technique, with virtually no blood loss. The patient was discharged without any complications and had a good postoperative recovery.

In another study by Pessoa et al. [23], the ExCITE technique was used for the extraction of a large fibroid (14.2 cm) from the abdominal cavity in the context of laparoscopic myomectomy: the specimen was efficiently extracted, providing aesthetic benefits for the patient. She was discharged on the second-day post-surgery without any complications. The study concluded that the extraction of large fibroids often poses a challenge for gynecological surgeons in the field of minimally invasive surgery. By using the ExCITE technique, a safe, agile and reproducible extraction can be performed, with efficient tissue removal, avoiding the use of electromechanical morcellators and the opening of the vaginal vault.

Furthermore, a similar use of the ExCITE technique was documented in a recent study by Adler et al. [24]: the patient, a fourteen-year-old girl with Mullerian agenesis, underwent laparoscopic surgery for the presence of bilateral uterine remnants, which were subsequently removed from the cavity using the ExCITE technique. She had an excellent postoperative recovery and was discharged on the same day, with minimal pain that did not require the use of pain medication. The study concluded that the ExCITE technique could be a good alternative to minilaparotomy in the removal of uterine remnants or other large samples in children, adolescents, and young adults following minimally invasive gynecological surgery.

As reported, the scant literature on the topic suggests that the ExCITE technique could be a choice for several reasons. It is a safe and reproducible technique that allows for contained tissue extraction. It minimizes the risk of endobag rupture as compared to mechanical morcellation inside the endobag with a laparoscopic morcellator [14]. By observing the patients enrolled in the study during the preoperative, intraoperative, and postoperative periods, we were able to compare the two techniques and determine that the ExCITE technique offers advantages not only for the surgeon but also for the patient. In particular, in Group A, we observed a reduction in postoperative pain on the VAS scale at 6 h and 12 h, which presumably correlates with shorter hospital stays. The ExCITE technique resulted in smaller scars for the patients, and 100% of them reported satisfaction with the treatment received. The study also allowed us to observe that the extraction times of the surgical specimens were shorter in Group A using the ExCITE technique, leading to a shorter overall operative time. Regarding blood loss and postoperative hemoglobin levels, the two techniques were comparable.

As supported by Brown et al. [25], despite the potential benefits of manual morcellation in an endobag, there is only one recent retrospective study about the outcomes of patients undergoing manual morcellation in an endobag during myomectomy [26]. Furthermore, to date, there are no prospective data to understand whether concerns related to intracorporeal mechanical morcellation are effectively reduced by manual morcellation in endobag. Many institutions have banned mechanical morcellation, but it is evident that there is a great need to focus on research in order to evaluate the intraoperative and postoperative outcomes of manual morcellation in endobag. Furthermore, it is important to collect data on outcomes, including histopathology of specimens, rates of endobag damage during the procedure, and short- and long-term complications, in order to provide appropriate recommendations on safer extraction techniques. The technique, although not recent, has been reassessed in our center because, as shown in this study, it allows achieving the same effectiveness in extracting the operative specimen from the abdominal cavity as compared to a minilaparotomy, with significant advantages for the patient in terms of postoperative pain and aesthetic outcomes.

Our study has several strengths: the ExCITE technique has demonstrated clear superiority in terms of postoperative pain, operative times, post-surgery recovery, and patient satisfaction as compared to minilaparotomy. In addition, the surgical times were shorter (both in terms of specimen extraction and overall operative times), allowing for cost containment.

The limitations include: ExCITE technique is not an innovative technique, although it is still relatively unknown; the sample size of the enrolled patients is not very large; there is an unequal distribution of patients in the two groups; the retrospective design of the study makes it difficult to state whether C-section is actually better than minilaparotomy. An important aspect to discuss is the difference between the two groups in terms of age, BMI and myoma size: regarding the age, surely a mininvasive approach such as ExCITE technique should be taken into account in young women where also the possibility of previous surgical scars should be lower than in older women; the significant difference in BMI would be explained in the surgeon's choice to avoid large abdominal incisions, avoiding the risk of post-surgical hematomas or infections in obese patients; eventually, the myoma size is a choice factor which of the two techniques to opt for, because the ExCITE technique for big myoma would nullify the advantages of minimally invasive surgery, especially in terms of surgical times. Anyway, we have specified that age and BMI were considered confounding factors.

## Conclusion

The ExCITE technique does not require specific training and allows the surgeon to offer a minimally invasive surgical option for patients, with also an aesthetic result. It is undoubtedly a safe and standardized approach that ensures tissue extraction without the need for mechanical morcellation, thereby avoiding intraperitoneal dissemination of tissue and preventing the development of benign conditions such as diffuse peritoneal leiomyomatosis or worse, malignancies, such as sarcoma. Further studies with large sample sizes are needed to confirm our data.

## Data Availability

Data are available upon request by the corresponding author.
